# The C-Terminal Domain of *Staphylococcus aureus* Zinc Transport Protein AdcA Binds Plasminogen and Factor H In Vitro

**DOI:** 10.3390/pathogens11020240

**Published:** 2022-02-12

**Authors:** Natália Salazar, Bruno Bernardi Yamamoto, Matilde Costa Lima de Souza, Ludmila Bezerra da Silva, Ana Paula Mattos Arêas, Angela Silva Barbosa

**Affiliations:** 1 Laboratory of Bacteriology, Instituto Butantan, São Paulo 05503-900, Brazil; natsalazar@gmail.com (N.S.); bbyamamoto@hotmail.com (B.B.Y.); matilde.souza@butantan.gov.br (M.C.L.d.S.); ludmilabs@gmail.com (L.B.d.S.); 2 Centro de Tecnologia de Vacinas (CT Vacinas), Federal University of Minas Gerais, Belo Horizonte 31270-901, Brazil; 3 Centro de Ciências Naturais e Humanas, Federal University of ABC (UFABC), Santo André 09210-580, Brazil

**Keywords:** *Staphylococcus aureus*, zinc transport protein AdcA, plasminogen, factor H, coagulation cascade, complement system

## Abstract

Bacterial acquisition of metals from a host is an essential attribute to facilitate survival and colonization within an infected organism. *Staphylococcus aureus*, a bacterial pathogen of medical importance, has evolved its strategies to acquire multiple metals, including iron, manganese, and zinc. Other important strategies for the colonization and infection of the host have been reported for staphylococci and include the expression of adhesins on the bacterial surface, as well as the acquisition of host plasminogen and complement regulatory proteins. Here we assess the ability of the zinc transport protein AdcA from *Staphylococcus aureus*, first characterized elsewhere as a zinc-binding protein of the ABC (ATP-binding cassette) transporters, to bind to host molecules. Like other staphylococcus ion-scavenging proteins, such as MntC, a manganese-binding protein, AdcA interacts with human plasminogen. Once activated, plasmin bound to AdcA cleaves fibrinogen and vitronectin. In addition, AdcA interacts with the human negative complement regulator factor H (FH). Plasminogen and FH have been shown to bind to distinct sites on the AdcA C-terminal portion. In conclusion, our in vitro data pave the way for future studies addressing the relevance of AdcA interactions with host molecules in vivo.

## 1. Introduction

*Staphylococcus aureus* is a relevant bacterial pathogen that causes significant morbidity and mortality, especially in a nosocomial environment, where antibiotic-resistant strains are common. The proliferation and full virulence of this pathogen is directly dependent on its ability to scavenge essential transition metals, which are normally chelated from host factors [1].

Transition metal ions, such as iron, manganese, and zinc, are crucial elements involved in diverse biochemical processes in bacterial cells. Zinc is one of the most abundant transition metals in live beings, since many bacterial enzymes, with a determined tridimensional structure, use zinc as a cofactor [2]. Additionally, some works have demonstrated that about 5% of bacterial proteomes are composed of zinc-interacting proteins [3]. Zinc is also important in a well-established single-species staphylococcal biofilm model, in which the SasG protein promotes cell–cell adhesion in a Zn^2+^-dependent manner [4].

Given the capacity to colonize different organs, pathogenic bacteria must deal with fluctuating transition metal concentrations in various host microenvironments [5]. Accurate control of bacterial zinc levels is mainly achieved by the regulation of Zn^2+^ uptake across membranes reviewed in [6,7]. *S. aureus* possesses two ABC (ATP-binding cassette) transport systems of zinc acquisition identified in the genomic data: CntABCDF and AdcABC, whose induction is associated with zinc limitation. While the CntABCDF system uses staphylopine, a chelating molecule similar to siderophores, to mediate the obtainment of the metal ion species [8,9], the AdcABC transporter, homologous to other bacterial ABC components, appears to utilize the classical mechanism of zinc uptake by direct binding to Zn^2+^ [9].

ABC transporters represent an important class of translocation systems found in bacteria. Typical ABC-type metal carrier complexes consist of three components: a metal-binding protein, also known as substrate-binding protein (SBP); an ATP-binding protein; and an integral membrane protein. The metallic ion translocation results from the interaction of the SBP—after ion uptake—and the integral membrane protein, a process driven by ATP hydrolysis [10,11,12].

Zinc-binding proteins in Gram-positive bacteria normally have two domains involved in metal coordination, while those found in Gram-negative bacteria usually have a single domain and additionally a zinc-binding auxiliary protein [13,14]. Studies suggest that the periplasmic protein ZinT in *Salmonella enterica* binds to ZnuA, the periplasmic component of the zinc transporter ZnuABC, and helps ZnuA in the process of zinc recruitment. Furthermore, analyses of ZinT alignment with the C-terminal AdcA portion of different Gram-positive bacteria show high homology and conservation of zinc-binding residues [13].

*Streptococcus* AdcA proteins possess two zinc-binding domains, and this architecture confers an advantage regarding zinc uptake. It has been demonstrated that zinc binds to the streptococcal AdcA N-terminal portion with high avidity, and as a consequence, the C-terminal local conformation is stabilized. This sequence of events ultimately culminates in an effective zinc transfer rate [15], which appears to provide clear benefits in hostile zinc scarce microenvironments, commonly encountered by the bacteria during infection. The *S. aureus* AdcA protein harbors two zinc-binding domains homologous to conserved metal-binding residues of streptococcal AdcA [9,13].

Our group previously demonstrated that MntC, belonging to the staphylococcal manganese ABC import system MntABC [16], possesses an additional function as an adhesin, whose targets include extracellular matrix components (ECMs) and coagulation cascade proteins [17]. The hypothesis of this present work relies on the assumption that similarly to MntC, AdcA would be able to display adhesive properties on ECM proteins and plasminogen with subsequent activation of plasmin, which could imply a probable mechanism of tissue invasion from a mucosal site, such as the nasopharynx. According to our data, AdcA binds with great avidity to human plasminogen and interacts with human factor H as well. Active plasmin, generated upon the addition of the plasminogen activator (uPA), degrades different substrates. However, unlike MntC, AdcA did not exhibit any property regarding the interaction with proteins from the host extracellular matrix.

## 2. Materials and Methods

### 2.1. Reagents, Purified Proteins, and Antibodies

The chromogenic substrate D-Val-Leu-Lys-r-nitroanilide dihydrochloride and ε-aminocaproic acid (EACA) were purchased from Sigma-Aldrich. All proteins and antibodies used in this study are listed in Table 1.

### 2.2. Cloning, Expression, and Purification of Recombinant AdcA (Full-Length Protein) and AdcA Fragments

The *AdcA* gene was amplified by PCR from the genomic DNA of the *S. aureus* ATCC 25923 strain. The primers used for the amplification of the sequence encoding the full-length protein and the N-terminal (nucleotides 64–528), intermediate (nucleotides 474–1005), and C-terminal (nucleotides 974–1551) fragments are presented in Table 2. Amplifications were performed with 100 ng of genomic DNA, 200 μM of each dNTP, 0.4 µM of each primer, 1.5 mM MgCl_2_, 1 × Taq buffer, and 2.5 U of Taq DNA polymerase (Thermo Fisher, Waltham, MA, USA) in a total volume of 50 μL. The mixtures were heated to 95 °C for 3 min, and reactions were cycled 30 × for 30 s at 95 °C, 30 s at 60 °C, and 1 min and 40 s at 72 °C. A final extension step was performed for 10 min at 72 °C. PCR fragments were cloned into a pGEM-T Easy vector (Promega, Madison, WI, USA) and subsequently transformed into competent *E. coli* DH5α cells. Following digestion with *BamHI* and *PstI* for the sequence encoding the full-length protein or *BamHI* and *NcoI* for the sequences correspondent to the N-terminal, intermediate, and C-terminal fragments, the inserts were subcloned into the pAE vector [18] for recombinant protein expression with an N-terminal 6XHis-tag. All constructs were analyzed by digestion with restriction enzymes besides DNA sequencing of amplicons, generated through the reaction with vector-specific primers. Expression of the recombinant proteins in mid-log-phase cultures of transformed *E. coli* BL21 (DE3) was achieved by the induction with 1 mM isopropyl-β-D-thiogalactopyranoside (IPTG) at 37 °C. After 3 h of incubation, bacterial cells were collected by centrifugation, and the cell pellets were resuspended in a solution composed of 20 mM sodium phosphate (pH 7.4) and 100 mM NaCl, followed by cellular lysis in a sonicator. The soluble and insoluble fractions were isolated by centrifugation at 26,000× *g* for 15 min. The His-tagged recombinant proteins were purified, from the supernatants, in Ni^2+^-charged chelating Sepharose columns (GE Healthcare, Buckinghamshire, UK). After adsorption of proteins, columns were sequentially washed with solutions containing 20 mM sodium phosphate (pH 7.4), 500 mM NaCl (buffer A), and increasing imidazole concentrations (5, 20, 40, and 60 mM). The proteins were eluted from the column with a solution comprising buffer A and 1 M imidazole. The eluates were slowly dialyzed against phosphate-buffered saline (PBS), followed by sample analysis utilizing a 12% sodium dodecyl sulfate–polyacrylamide gel electrophoresis (12% SDS-PAGE). Protein concentrations were estimated using the Bradford method (Pierce, Thermo Fisher Scientific Inc., Rockford, IL, USA), according to the manufacturer’s instructions. Control proteins (MntC from *Staphylococcus aureus* and LigA and LigB from *Leptospira interrogans*) were obtained as previously described [17,19].

### 2.3. Antiserum against Recombinant AdcA

The protocol for obtention of antiserum against recombinant AdcA was approved by the Committee on Ethics of Instituto Butantan (# 9452010316, meeting of 16 March 2016), which in turn is in accordance with Law 11.794, published on 8 October 2008; Decree 6899, made public on 15 July 2009; and the rules issued by the National Council for the Control of Animal Experimentation (CONCEA). A healthy 60-day-old female New Zealand rabbit was immunized intramuscularly with 100 µg of recombinant protein using 2.5 mg of aluminum hydroxide as adjuvant (total volume of 500 µL). Two subsequent booster injections were given at 15-day intervals. The animal was bled from the marginal ear vein prior to immunizations (preimmune bleeding/serum control) and 2 weeks after the third dose. Total bleeding was performed by cardiac puncture under deep terminal anesthesia with ketamine (50 mg/kg) and xylazine (3 mg/kg). The blood was centrifuged for 5 min at 3000× *g*, and the serum was stored at −20 °C. Antibody titers were determined by enzyme-linked immunosorbent assay (ELISA). Briefly, 96-well plates (high binding, Costar, Corning Inc., Corning, NY, USA) were coated with 1.0 μg/well of purified AdcA and then washed. After blocking with 5% skimmed milk, rabbit serum samples were serially diluted 2-fold beginning with a 1:50 dilution; plates were incubated for 1 h at 37 °C, and then washed. Horseradish peroxidase-conjugated anti-rabbit IgG antibodies (Sigma-Aldrich, St. Louis, MO, USA) were added to the wells. The plates were incubated for an additional hour, and the wells were washed three times. After that, o-phenylenediamine 0.04% (*w*/*v*) in citrate phosphate buffer (pH 5.0) plus 0.01% H_2_O_2_ was added to the plates. The reaction proceeded for 15 min, and 50 μL of 8 M H_2_SO_4_ was added to yield a chromogenic product. An absorbance at 492 nm was determined in a microplate spectrophotometer (Multiskan EX, Labsystems Uniscience, Waltham, MA, USA).

### 2.4. Binding of AdcA to Plasminogen

ELISA plate wells (high binding, Costar, Corning Inc., Corning, NY, USA) were coated with 1 µg of plasminogen in 100 µL of PBS. After incubation at 4 °C for 16–20 h, the wells were washed thrice with PBS–0.05% Tween 20 (PBS-T) and then blocked with 200 µL of 1% gelatin for 2 h at 37 °C. To assess the dose-dependent attachment of AdcA to plasminogen, protein concentrations varying from 0 to 1 µM were added per well in 100 µL of PBS. After 1.5 h at 37 °C, six washes with PBS-T were performed, followed by the detection of bound AdcA using 100 μL of a 1:5000 dilution of polyclonal specific antiserum produced in rabbit. Incubation proceeded for 1 h, and following three washes with PBS-T, the wells were allowed to incubate for 1 h with 100 μL of a 1:5000 dilution of peroxidase-conjugated goat anti-rabbit immunoglobulin G (IgG) antibody (Sigma-Aldrich, St. Louis, MO, USA) in PBS. All incubations occurred at 37 °C. After washing the wells thrice with PBS-T, the reactions were developed as mentioned above. BSA was used as negative control.

### 2.5. Effects of Ionic Strength and the Role of Lysines in AdcA–Plasminogen Interactions

The role of lysines and ionic strength in AdcA plasminogen interactions was assessed essentially as mentioned above, with some adapted conditions from the procedure described in [17]. Briefly, ELISA plate wells, coated with plasminogen (10 µg/mL), were incubated with AdcA (10 µg/mL) plus ε-aminocaproic acid (0–10 mM) or AdcA (10 µg/mL) plus NaCl (0–800 mM). Bound AdcA was detected as mentioned in the previous section. All experiments were performed in triplicate and repeated, at least, twice. Statistical analysis was performed by using Student’s two-tailed *t*-test, in which a *p*-value of less than 0.05 was considered statistically significant.

### 2.6. Plasminogen Activation

Plasminogen activation was assessed as previously described [17]. In brief, microtiter plate wells were coated with 10 µg/mL of AdcA, MntC (positive control), or BSA (negative control) for 16–20 h at 37 °C. After blockage with 3% BSA, plasminogen, at a final concentration of 20 µg/mL, was added. After an incubation of 1 h at 37 °C, the wells were washed thrice with PBS-T, followed by the addition of the human urokinase-type plasminogen activator (uPA, Sigma-Aldrich) (3 U/well) plus the chromogenic substrate D-valyl-leucyl-lysine-r-nitroanilide dihydrochloride (25 µg/well, Sigma-Aldrich), dissolved in PBS. The plates were incubated at 37 °C for 24 h, when the absorbance at 405 nm was determined.

### 2.7. Degradation of Fibrinogen and Vitronectin by Plasmin Bound to AdcA

Recombinant AdcA (10 µg/mL) and BSA (10 µg/mL) were immobilized onto microtiter plate wells for 16–20 h at 37 °C and incubated with plasminogen (20 µg/mL), as described above. After washing the wells three times with PBS-T, human fibrinogen or vitronectin (500 ng), in combination with plasminogen activator uPA (3 U), was added. Reaction components, previously incubated for up to 4 h at 37 °C, underwent separation by 12% SDS-PAGE and transference to nitrocellulose membranes. Products of fibrinogen or vitronectin degradation were detected by Western blotting with anti-human fibrinogen or anti-human vitronectin (1:5000) antibodies, both produced in rabbit. This step was followed by an incubation with a 1:10,000 dilution of peroxidase-conjugated goat anti-rabbit IgG antibody (Sigma-Aldrich). Positive signals were revealed by detection of enhanced chemiluminescence (West Pico, Pierce).

### 2.8. Far Western Blot

Recombinant proteins—full-length AdcA and its fragments—were subjected to separation in a 12% SDS-PAGE, followed by transference to nitrocellulose membranes. The immobilized proteins were then incubated for 1.5 h with 50 µg of purified plasminogen or factor H, both diluted in PBS. After five washes with PBS-T, the membranes were incubated with rabbit anti-human plasminogen (1:3000) or goat anti-human factor H (1:5000) antibodies, followed by appropriate secondary peroxidase-conjugated antibodies (1:10,000). Positive signals were detected by enhanced chemiluminescence (West Pico, Pierce). Purified plasminogen and BSA were included as positive and negative controls, respectively, in plasminogen experiments, whereas LigA and LigB (two surface-exposed *Leptospira* proteins) were included as positive controls for FH binding [20].

### 2.9. Competition Assays

In order to assess whether plasminogen and FH compete for the same binding site(s) on AdcA, the recombinant protein—100 µL at a final concentration of 10 µg/mL—was immobilized on ELISA plate wells, as described above. A blockage with 200 µL of 1% gelatin, for 2 h at 37 °C, was followed by the addition of 1 microgram of FH, mixed with different amounts of plasminogen according to FH/plasminogen molar ratios varying from 1:0.25 to 1:4. The assay was accomplished in duplicate, in a way that the AdcA–FH and AdcA–plasminogen complexes could be simultaneously detected in our system. In order to achieve this premise, proteins were incubated with anti-human plasminogen (1:3000) and anti-human FH antibodies (1:5000), followed by interaction with secondary peroxidase-conjugated antibodies (1:5000), as described above. Three independent experiments were performed, each one in duplicate.

## 3. Results

### 3.1. AdcA Sequence Analysis

Multiple sequence alignment analysis of AdcA with its counterparts from *Streptococcus pneumoniae*, *Streptococcus suis*, *Enterococcus faecalis*, and *Staphylococcus haemolyticus* and with the single-domain protein ZnuA from *Salmonella enterica* and ZinT from *Salmonella enterica*, *Escherichia coli*, and *Bacillus subtilis* was performed with Clustal Omega (https://www.ebi.ac.uk/Tools/msa/clustalo/ (accessed on 20 December 2021). The *S. aureus* AdcA N-terminal domain harbors the conserved residues involved in metal binding, also present in the four above-mentioned double-domain proteins and the single-domain ZnuA: His^67^, His^154^, His^218^, and Glu^292^, besides the His-rich region observed in the zinc-binding SBP family (Supplementary Figure S1). The AdcA C-terminal portion harbors three additional histidines (His^468^, His^477^, His^479^), also related to metal binding and present in other double-domain and ZinT proteins analyzed [13].

### 3.2. Expression and Purification of Full-Length AdcA and Its Fragments

**Figure 1:**
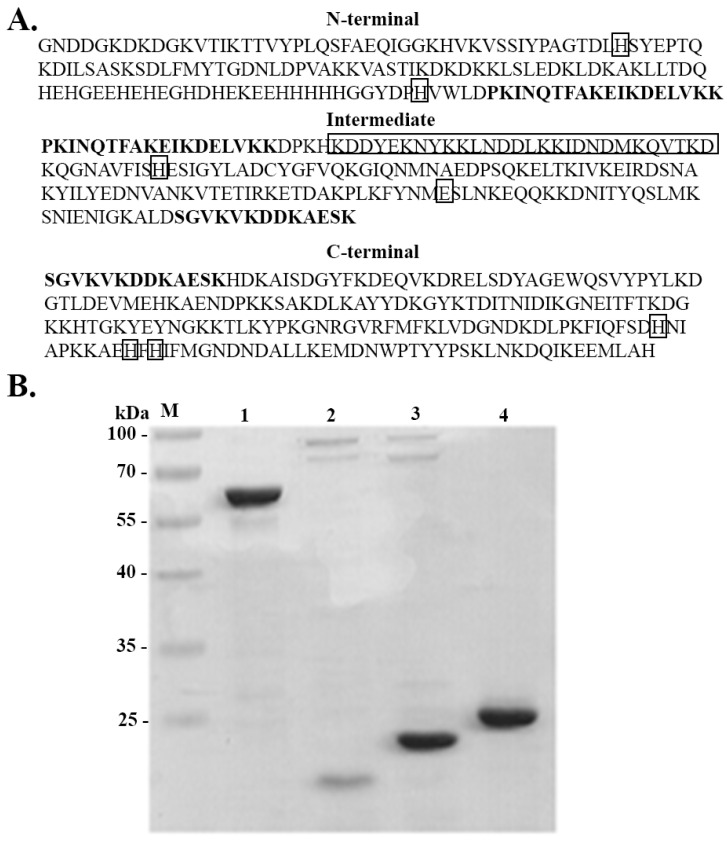
Protein sequence of AdcA domains and purification of recombinant proteins. (**A**) AdcA N-terminal (amino acids 22 to 176), intermediate (amino acids 159 to 335), and C-terminal (amino acids 323 to 516) domains are shown. The overlapping aminoacids in the N-terminal and intermediate and in the intermediate and C-terminal fragments are in bold. The coiled-coil domain in the intermediate fragment and the histidines involved in metal-binding are shown in boxes. (**B**) Twelve percent SDS-PAGE showing full-length AdcA (1), AdcA N-terminal (2), intermediate (3), and C-terminal (4) fragments. Molecular mass marker (M).

In order to identify functional domains of AdcA, three recombinant fragments were produced. The criterion to split the sequence was based on the size of the fragments, and we aimed to maintain structural and functional specificities. The residues involved in metal binding are present in all three fragments: the N-terminal contains the residues His^67^ and His^154^ and the conserved His-rich region, the intermediate fragment contains a coiled-coil domain and the residues His^218^ and Glu^292^, and the C-terminal portion of AdcA harbors the histidines of the cited ZinT proteins [13]. All fragments have overlapping regions, highlighted in bold (Figure 1A). Purified full-length AdcA and its recombinant fragments appear as single major bands in SDS-PAGE, and the observed mobilities correspond to their calculated molecular masses: 59 kDa (full-length AdcA), 19 kDa (N-terminal fragment), 22 kDa (intermediate fragment), and 24 kDa (C-terminal fragment) (Figure 1B).

### 3.3. AdcA Interacts with Plasminogen

**Figure 2:**
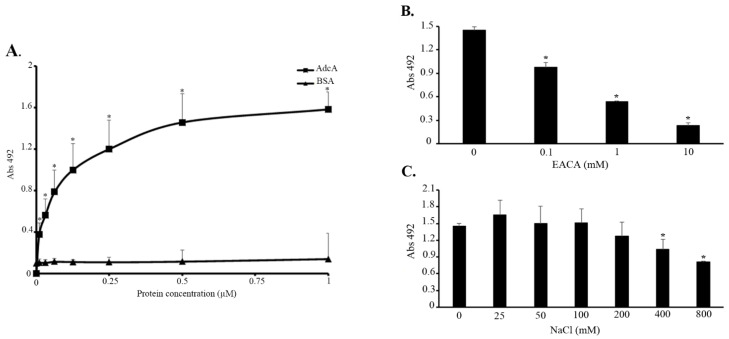
AdcA binding to plasminogen and the role of lysine residues or NaCl in this interaction. (**A**) Binding of AdcA to plasminogen as a function of protein concentration. Increasing amounts of AdcA (0.016, 0.031, 0.062, 0.125, 0.25, 0.5, 1 μM) were incubated with immobilized plasminogen or BSA (10 μg/mL). Bound AdcA was detected with polyclonal anti-AdcA serum, followed by peroxidase-conjugated secondary antibodies. (**B**) Role of lysines and (**C**) salt in AdcA/plasminogen interactions. AdcA (10 μg/mL) was added to plasminogen-coated wells in the presence of ε-aminocaproic acid (0.1–10 mM) or NaCl (25–800 mM). AdcA–plasminogen complexes were detected with polyclonal anti-AdcA serum, followed by peroxidase-conjugated secondary antibodies. Data show the mean absorbance value at 492 nm ± the standard deviation of three independent experiments, with correspondent duplicates. For this analysis, a Student’s *t*-test was used, in which * *p* < 0.05 was considered statistically significant.

In a previous work, we demonstrated that MntC, a *S. aureus* manganese transport protein, is capable of binding to several extracellular matrix and coagulation cascade components, including laminin, collagen type IV, cellular and plasma fibronectin, plasminogen, and fibrinogen [17]. This prompted us to evaluate whether AdcA, a zinc transport protein, would also display binding properties. The interaction of AdcA with the above-mentioned host components was then investigated, and a dose-dependent and saturable binding to plasminogen was observed (Figure 2A). The apparent *K_D_* of this interaction was calculated in GraphPad Prism using the best fit to total binding saturation function. In these adjustment conditions, the same order of magnitude for AdcA (*K_D_* = 45.09 nM) and MntC, used as a control (*K_D_* = 14.75 nM) was observed. No significant binding activity to other host molecules, including laminin, collagen type IV, cellular and plasma fibronectin, and fibrinogen, was detected (data not shown).

The kringle domains of plasminogen may interact with lysine residues present on diverse receptors [21]. Given the high content of such residues in AdcA (16%), we assessed whether AdcA–plasminogen interactions would be affected by the presence of lysine analog ε-aminocaproic acid. As depicted in Figure 2B, EACA markedly inhibited plasminogen–AdcA interactions in a dose-dependent manner, suggesting that the lysine-binding sites on the kringle domains of plasminogen may be involved in the event. The role of ionic strength in AdcA–plasminogen interactions was also evaluated. Salt concentrations greater than 400 mM significantly decreased AdcA binding to its target (Figure 2C). It appears that competitive ionic or ion–dipole interactions with lysine residues of AdcA might occur in higher ionic strength.

### 3.4. AdcA-Bound Plasminogen Is Activated to Plasmin

**Figure 3:**
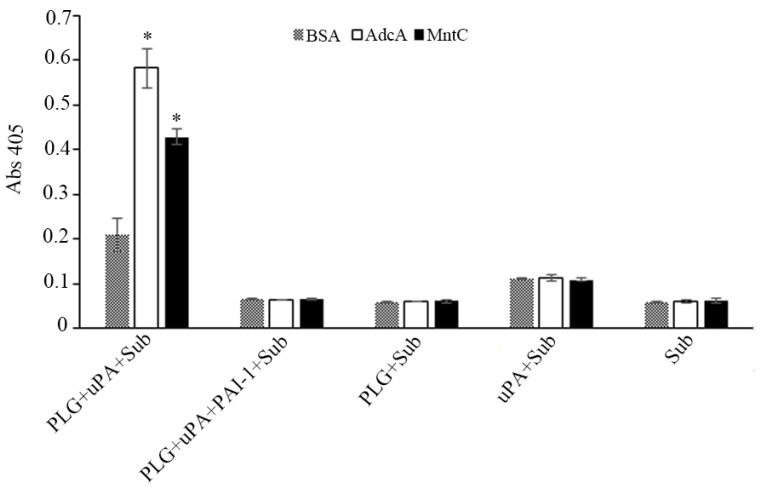
Active plasmin is produced from AdcA-bound plasminogen. Microtiter plate wells, previously sensitized with recombinant proteins or BSA (10 µg/mL), were incubated with plasminogen (20 µg/mL). The wells were washed with PBS-T, and plasmin activity was assessed upon addition of the chromogenic substrate D-valyl-leucyl-lysine-r-nitroanilide dihydrochloride (Sub) (25 µg/well) and the plasminogen activator uPA (3 U)). Control reactions: (PLG + uPA + PAI-1 + Sub)—plasminogen activator inhibitor 1 (PAI-1), which inhibits uPA, was added along with uPA and Sub; (PLG + Sub)—uPA was omitted, preventing the conversion of plasminogen to plasmin; (uPA + Sub)—plasminogen was omitted, preventing substrate cleavage; (Sub)—both uPA and plasminogen were omitted. Data show the mean absorbance value at 405 nm ± the standard deviation of three independent experiments, with correspondent duplicates. For this analysis, a Student’s *t*-test was used, in which * *p* < 0.05 was considered statistically significant. PAI-1 is the plasminogen activator inhibitor 1.

In vivo, after binding to its receptors, plasminogen is converted to its active form, plasmin, by plasminogen activators (uPA, tPA) [22]. As shown in Figure 3, plasminogen could be activated by uPA once bound to immobilized AdcA. Consequently, the freshly generated plasmin was able to cleave the chromogenic substrate D-valyl-leucyl-lysine-r-nitroanilide dihydrochloride (Sub). *S. aureus* MntC, previously shown to be a plasminogen-binding protein [17], was included as a positive control, and BSA as a negative one. No detectable cleavage of the chromogenic substrate was disclosed in the presence of plasminogen activator inhibitor 1 (PAI-1) or in the absence of uPA, plasminogen, or both uPA and plasminogen, regardless of the protein used for coating (BSA, AdcA, or MntC) (Figure 3).

### 3.5. Plasmin Bound to AdcA Cleaves Fibrinogen and Vitronectin

**Figure 4:**
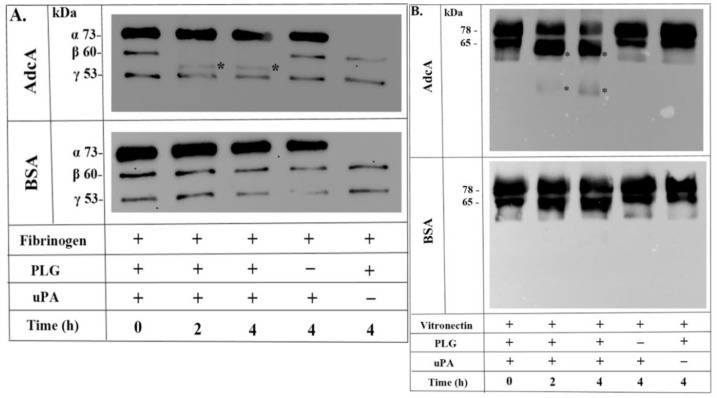
Immobilized AdcA-plasmin(ogen) degrades human fibrinogen and vitronectin. Microtiter plate wells, previously sensitized with recombinant proteins or BSA (10 µg/mL), were incubated with plasminogen (20 µg/mL). After washing, an additional incubation with fibrinogen (500 ng) (**A**) or vitronectin (500 ng) (**B**) plus uPA (3 U) took place until the specified time points. Samples underwent separation by SDS-PAGE and transference to nitrocellulose membranes. Membranes were incubated with anti-human fibrinogen (**A**) or anti-human vitronectin antibodies, (**B**) followed by the secondary HRP-conjugated antibodies. Controls excluding uPA or plasminogen were included. Cleavage products are indicated by asterisks (*).

It is well established that active plasmin has a variety of physiological functions, playing a decisive role in fibrinolysis, wound healing, tissue remodeling, inflammation, and ECM degradation (for a recent review, see [23]). In the present study, we assessed the degradation of human fibrinogen and vitronectin by plasmin(ogen) bound to immobilized AdcA. According to our results, AdcA-bound plasmin degraded the fibrinogen β-chain after 2 h of incubation (Figure 4A) and time-dependently degraded human vitronectin, generating 61–63 and 35–36 kDa cleavage fragments (Figure 4B). No cleavage products were observed in the absence of the substrates or uPA (Figure 4A,B). BSA was used as a negative control, and no degradation products were observed in this condition as well (Figure 4A,B). It is worth mentioning that uPA comigrates with fibrinogen α-chain, which migrates as a 73 kDa band, but is not visible in Figure 4A, lane 5 (this lane contains plasminogen and fibrinogen, but not uPA).

### 3.6. AdcA Interacts with Plasminogen and FH through Its C-Terminal Domain

**Figure 5:**
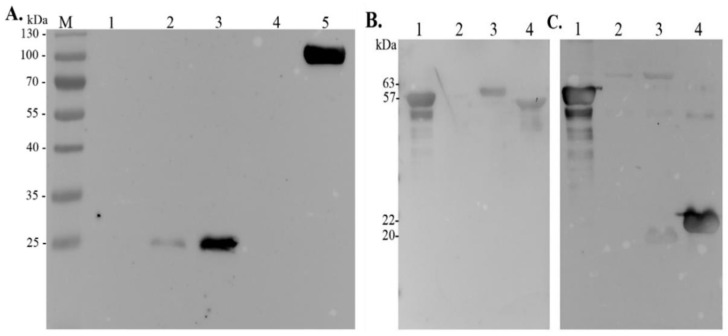
AdcA interacts with plasminogen and FH through its C-terminal region. (**A**) AdcA N-terminal fragment (1), AdcA intermediate fragment (2), AdcA C-terminal fragment (3), BSA (4), and purified plasminogen (5) were subjected to SDS-PAGE and transferred to a nitrocellulose membrane. The membrane was incubated with purified plasminogen (50 μg) and probed with anti-human plasminogen antibody, followed by the addition of corresponding secondary HRP-conjugated antibodies. (**B**) Full-length AdcA (1), BSA (2), LigA (3), and LigB (4) were subjected to SDS-PAGE and transferred to a nitrocellulose membrane. The membrane was incubated with purified factor H (50 μg) and probed with anti-human FH antibody, followed by the addition of corresponding secondary HRP-conjugated antibodies. (**C**) Full-length AdcA (1), AdcA N-terminal fragment (2), AdcA intermediate fragment (3), and AdcA C-terminal fragment (4) were incubated with purified human FH and probed with anti-human FH antibody as described above.

With the aim of mapping the AdcA region(s) responsible for plasminogen-binding activity, a ligand affinity blot was performed with the three AdcA fragments shown in Figure 1. The C-terminal fragment was the only region that interacted with plasminogen, revealing that the binding capacity of AdcA to plasminogen is conferred by this portion (Figure 5A), which contains the three histidines involved in zinc binding, also found in the double-domain streptococcal AdcA proteins. Purified plasminogen was included as a positive control, and BSA was used as a negative one.

Considering that staphylococci employ diverse strategies to avoid innate immune responses, such as complement deposition, and given that several plasminogen-binding proteins not infrequently are additionally able to interact with factor H, we evaluated the interaction of *Staphylococcus* AdcA with this important complement regulatory protein (factor H). The capacity of full-length AdcA to bind to FH was initially examined. For this assay, *Leptospira* LigA and LigB were used as positive controls [19]. As seen in Figure 5B, AdcA interacted with FH, and binding was mostly mediated by its C-terminal portion (Figure 5C).

### 3.7. Plasminogen and FH Do Not Share Binding Sites on AdcA

**Figure 6:**
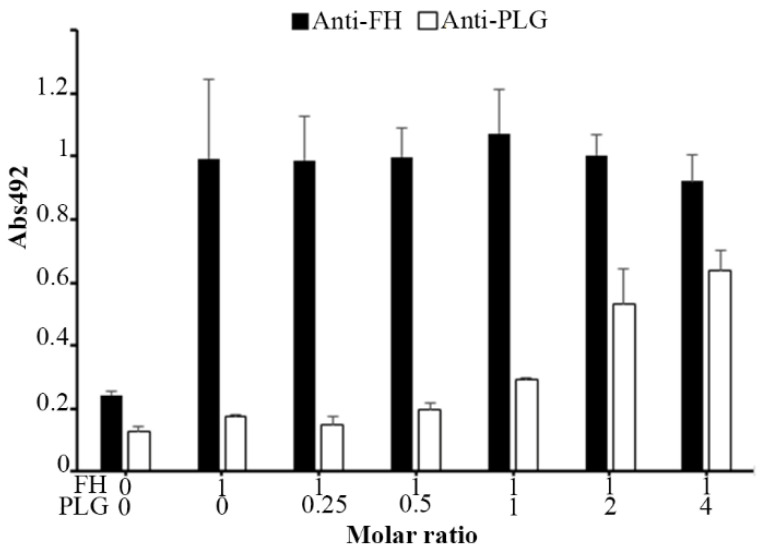
Plasminogen and FH bind simultaneously to AdcA. AdcA (10 µg/mL) immobilized on a microtiter plate was incubated with a fixed amount of FH (1 µg) mixed with different amounts of plasminogen (0 to 4 μg). Bound proteins were detected with either anti-plasminogen or anti-FH polyclonal antibodies. The assay was performed in duplicate. Each point represents the mean absorbance value at 492 nm ± the standard deviation of three independent experiments.

Given that both plasminogen and FH interact with the C-terminal portion of AdcA, we then assessed whether they compete for the same binding sites. For this, a fixed amount of FH (1 µg) and increasing quantities of plasminogen (0–4 µg) were added to the immobilized recombinant protein. Plasminogen and FH were independently detected with specific antibodies. As FH interaction with AdcA was not affected by the presence of increasing amounts of plasminogen, we conclude that plasminogen and FH do not have overlapping binding sites on AdcA (Figure 6).

## 4. Discussion

Understanding the mechanisms of the pathogenicity of *S. aureus* is essential for the development of more effective therapies in order to prevent and eliminate infections caused by this bacterium. *S. aureus* survival and virulence in the host depend on the bacterial ability to acquire essential metals, especially iron, manganese, and zinc, normally scarce in the infection sites [1].

Zinc homeostasis is controlled by a delicate balance to maintain stable levels of intracellular transition metal [7]. Although an excess of this metallic ion would compromise the perfect functioning of cell machinery, adequate concentration of zinc is a prerequisite for the full activity of many enzymes and transcription factors, as well as for bacterial metabolism, and for anti-inflammatory processes responsive to immune actions triggered by the host. In this work, we describe a new function for the zinc-binding surface protein of *S. aureus* AdcA, a member of the metal-binding receptor family (MBR). Unlike other MBRs, the *adcA* gene is not part of an ABC transporter operon, raising the hypothesis that AdcA may share the integral membrane protein and the cytoplasmic ATPase with another staphylococcal metal-binding receptor. Homologous *S. pyogenes* AdcA and *S. pneumoniae* AdcA share 61% of identical residues, and sequence alignment analysis of these proteins with *S. aureus* AdcA revealed a 37% degree of similarity. Most importantly, *S. aureus* AdcA conserves the histidine residues in both N- and C-termini, besides the amino acids aspartate or glutamate present in the C-terminal portion, collectively involved in metal binding. This array of amino acids is the fingerprint of zinc-interacting proteins [24], especially metal-binding receptor family members.

In addition to nutrient uptake functions, such as transition metals, proteins present on the surface of *S. aureus* play crucial roles in colonization, the establishment of infection, and immune evasion by interacting with host molecules of the uppermost cellular layer, extracellular matrix, and complement system. Well-characterized adhesins of *S. aureus* belong to the MSCRAMM (microbial surface components recognizing adhesive matrix molecules) family [25,26]. These family members harbor a conserved C-terminal cell wall sorting signal containing a Leu-Pro-X-Thr-Gly (LPXTG) motif, which is the target of an enzymatic factor, sortase A, involved in surface protein anchoring to membrane [27]. Examples of MSCRAMMs include clumping factors A (ClfA) and B (ClfB) responsible for fibrinogen binding, fibronectin-binding proteins A (FnBPA) and B (FnBPB) (reviewed in [28]), and collagen adhesin (Cna) [29].

Interaction of bacterial pathogens with the host’s plasminogen and its conversion to plasmin allows bacteria to invade the host by cleaving tissue components and fibrin clots and escaping the host’s defenses by removing IgG and C3b opsonins [30]. Plasminogen can be captured by the cell surface of *S. aureus* where the zymogen can be activated by staphylokinase or host urokinase and t-PA [31]. The binding of multifunctional proteins anchored and not anchored to the surface of *S. aureus* to plasminogen has been described. Enolase, triosephosphate isomerase (TPI), and GAPDH, which are proteins of the glycolytic pathway, can migrate to the surface of *S. aureus* and interact with plasminogen [32]. Plasmin, the active form of plasminogen with serine protease activity, has in its structure five triple-disulfide-bonded kringle domains containing lysine-binding sites (LBSs) [33]. C-terminal lysyl residues mediate the binding of different receptors to plasminogen, including α-enolase of *S. pneumoniae* and *S. pyogenes* [34,35].

In this work, we demonstrated that AdcA from *S. aureus* binds human plasminogen in a dose-dependent and saturable manner. The magnitude of apparent *K_D_* values encountered for the protein fell within the nanomolar range, thus indicating that they bind to plasminogen with high affinity. Based on these findings, we can anticipate that AdcA may act as a *S. aureus* virulence factor by recruiting plasminogen to the staphylococcal surface, which would be engaged in triggering a sequence of events necessary for bacteria spread. Blockage of plasminogen lysine-binding sites by EACA and incubation of the two proteins under high salt concentrations significantly inhibited AdcA–plasminogen interactions, pointing to a role of lysine residues and adequate ionic strength in AdcA binding to plasminogen. Active plasmin was generated from AdcA-bound plasminogen in the presence of uPA, as attested by the proteolytic cleavage of a synthetic chromogenic plasmin substrate. It is well known that plasmin has a variety of physiological functions, playing a crucial role in fibrinolysis, wound healing, tissue remodeling, inflammation, and ECM degradation (for a recent review, see [23]). Pathogenic bacteria take advantage of this host mechanism to gain access to deeper tissues, for instance, lungs, blood, heart, or even bones. Their journey, quite often, initiates in colonizing mucosal sites, such as the nasopharynx, where bacterial species live as commensals until they capture all nutrients the invasion demands. In this stage, the infection episodes are usually asymptomatic. The existence of carriers in these conditions constitutes a great challenge to epidemiological vigilance and transmission control [36].

In this work, further evidence of plasmin activity was provided by the degradation of the physiological substrates—fibrinogen and vitronectin—by plasmin bound to immobilized AdcA. Complete degradation of human fibrinogen β-chain occurred, which displayed a profile similar to the one observed for fibrinogen cleavage by MntC-bound plasmin(ogen) [17]. Degradation fragments for human vitronectin were also observed, and the cleavage pattern seems to be compatible with the expected profile of vitronectin plasminolysis. According to Kost et al. (1996) [37], the proteolytic action of plasmin on vitronectin first produces 61–63 kDa fragments upon removal of C-terminal residues of the molecule, followed by a second round of cleavage, which generates 35–56 kDa fractions as a consequence of the N-terminal vitronectin processing.

The AdcA region(s) involved in plasminogen binding were assessed, and according to our data, this interaction is clearly mediated by the AdcA C-terminal region. Defining the precise location of lysines involved in plasmin(ogen) binding is still a perspective for a future work. Our results provide evidence that lysine residues play a relevant role in plasmin(ogen)–AdcA interactions. However, whether additional residues would contribute to this binding remains uncertain. It is suggested that positively charged residues — not only lysines — in a hydrophobic environment may contribute to plasminogen binding in an effective way (reviewed in [21]). Staphylococci are highly efficient bacteria, equipped with multiple strategies to infect and persist in the host. It is worth mentioning that *S. aureus*, in particular, expresses multifunctional surface-exposed proteins that have been shown to interact with two or more plasma proteins simultaneously, including complement regulatory proteins and plasminogen (reviewed in [38]). In this context, AdcA also interacted with human factor H, an important soluble regulator of the alternative pathway of the complement system. Acquisition of FH by pathogenic bacteria is crucial for survival in the bloodstream during infection, and *S. aureus* has been shown to express several FH-binding proteins, such as Efb, Sbi, and SdrE [39,40,41].

In conclusion, in this work we provide evidence of a novel function for *S. aureus* AdcA. Besides contributing to zinc homeostasis, AdcA may help this important human pathogen to invade and colonize the human host by acquiring plasminogen and the negative complement regulator FH. Although our data support the hypothesis that AdcA is a relevant *S. aureus* virulence factor, it remains to be investigated whether AdcA may serve, like MntC, as a component for a vaccine formulation against *Staphylococcus* infections.

## Figures and Tables

**Table 1:** Proteins and antibodies used in this study.

Protein	Origin	Molecular Weight (kDa)	UniProtKBs
AdcA (*S. aureus*)	This study	59.1	A0A0D3QA93
AdcA N-terminal fragment (*S. aureus*)	This study	19	-
AdcA intermediate fragment (*S. aureus*)	This study	22	-
AdcA C-terminal fragment (*S. aureus*)	This study	24	-
MntC	Salazar et al., 2014	35	Q8VQS9
LigA C-terminal fragment	Castiblanco-Valencia et al., 2016	63	-
LigB C-terminal fragment	Castiblanco-Valencia et al., 2016	56	-
Human plasminogen	Commercial (Sigma-Aldrich)	92	P00747
	Commercial (Sigma-Aldrich)	α-73	α-P02671
Human fibrinogen		β-60	β-P02675
		γ-53	γ-P02679
Human vitronectin	Commercial (Sigma-Aldrich)	75/65	P04004
Human complement factor H	Commercial (Complement Technology)	155	Q03591
Urokinase-type plasminogen activator	Commercial (Sigma-Aldrich)	46.9	P00749
Plasminogen activator inhibitor 1	Commercial (Sigma-Aldrich)	43.4	P05121
Goat anti-human factor H	Commercial (Quidel)	-	-
Rabbit anti-human fibrinogen	Commercial (Calbiochem)	-	-
Rabbit anti-human plasminogen	Commercial (Sigma-Aldrich)	-	-
Rabbit anti-human vitronectin	Commercial (Complement Technology)	-	-
Peroxidase-conjugated anti-goat immunoglobulin G	Commercial (Sigma-Aldrich)	-	-
Peroxidase-conjugated goat anti-rabbit immunoglobulin G	Commercial (Sigma-Aldrich)	-	-
Bovine serum albumin	Commercial (Sigma-Aldrich)	69.2	P02769

**Table 2:** Primers used for the amplification of the sequences encoding the full-length AdcA protein and its fragments. The restriction sites are highlighted in bold.

Full-length AdcA	F: 5′-***GGATCC***GGGAATGATGATGGAAAAGATAAAGATGGC-3′
	R: 5′-***CTGCAG***TTAATGCGCTAACATTTCTTCTTTG-3′
N-terminal fragment	F: 5′-***GGATCC***GGGAATGATGATGGAAAAGATAAAGATGGC-3′
	R: 5′***-CCATGG***TTATTTTTTCACTAATTCATCTTTAATTTCTTTAGCG-3′
Intermediate fragment	F: 5′-***GGATCC***CCTAAAATTAACCAAACTTTCGCTAAAG-3′
	R: 5′-***CCATGG***TTATTTACTTTCAGCTTTGTCGTCTTTCAC-3′
C-terminal fragment	F: 5′-***GGATCC***AGTGGTGTTAAAGTGAAAGACGAC-3′
	R: 5′-***CCATGG***TTAATGCGCTAACATTTCTTCTTTG-3′

## Data Availability

Data is contained within the article or Supplementary Material.

## Supplementary Materials

The following supporting information can be downloaded at: https://www.mdpi.com/article/10.3390/pathogens11020240/s1, Figure S1: Sequence alignment of AdcA and other zinc-binding proteins.
