# Supplementary material for: The C-Terminal Domain of Staphylococcus aureus Zinc Transport Protein AdcA Binds Plasminogen and Factor H In Vitro
Source: Pathogens. 2022 Feb 12;11(2):240. doi: 10.3390/pathogens11020240 (PMC8878332; doi:10.3390/pathogens11020240)
Supplement: Supplementary file 1 [file pathogens-11-00240-s001.zip › pathogens-1544789-supplementary.pdf]

Supplementary Figure S1

Sequence alignment of AdcA and other zinc-binding proteins. Multiple sequence alignment of *Staphylococcus aureus, Streptococcus pneumoniae, Streptococcus suis, Enterococcus faecalis and Staphylococcus haemolyticus* AdcA two zinc-binding domain proteins was performed with Clustal Omega (https://www.ebi.ac.uk/Tools/msa/clustalo/). Conserved residues of N-terminal (His^{67}, His^{154}, His^{218}, Glu^{292}) and C-terminal (His^{468}, His^{477}, His^{479}) domains involved in metal binding are shown in boxes.

An * (asterisk) indicates positions which have a single, fully conserved residue, A : (colon) indicates conservation between groups of strongly similar properties, A . (dot) semi-conserved substitutions

```
S.aureus            MKKKLGMLLLVPAVTLSLAACGNDDGKDK----DGKVTIKTTVYPLQSFAEQIGGKHVKV  56
S.haemolyticus      MKRFLSLTVLLSIILCVLVACGKEDSSNKASKDGDKINVSTTVYPLQSFIEQIGGNHVNV  60
E.faecalis          -MKKFTLPLLAALSLILFGACGKTNTSDKTADGKEKLSIVTTFYPMYDFTKNIVGDEGDV  59
S.pneumoniae        -MKKISLL-LASLCALFLVACSNQ-----K-QADGKLNIVTTFYPVYEFTKQVAGDTANV  52
S.suis              -MKKVGLL-FLSVSALLLGACSNS-----TASEDGKLDIVTTFYPVYEFTKQVAGDEANV  53
                      : . :  :       : **.:            *: : **.**: .* ::: *.  .*

S.aureus            SSIYPAGTDLHSYEPTQKDILSASKSDLFMYTGDNLDPVAKKVASTIKDKDKKLSLEDKL  116
S.haemolyticus      SSIYPAGSDLHDYEPTQKDMLKVNKSDLFVYTGDDLDPVAKKVAATIKDDKKKVSLQDKL  120
E.faecalis          KLLIPAGSEPHDYEPSAKDMATIHDADVFVYHNENMESWVPKAAKGWKKGAPNV--IKGT  117
S.pneumoniae        ELLIGAGTEPHEYEPSAKAVAKIQDADTFVYENENMETWVPKLLDTLDKKKVKT--IKAT  110
S.suis              DLLVKAGTEVHGYEPSAKDIARIQEADAFVYENENMETWVHDVEKSLDTTKVNV--ISAT  111
                    . :  **:: * ***: * :    .:* *:* .::::  . .     .    :    .

S.aureus            DKAKLLTDQHEHGEEHEHEGHDHEKEEHHHHHGGYDPHVWLDPKINQTFAKEIKDELVKK  176
S.haemolyticus      DRSTLLTDQHEHGDEEHAD----AHEHHHHHHGGYDPHVWLDPEKNKIFAKEIKEQLVAK  176
E.faecalis          ENMVLLPGSDEDGHDH-----DHEHGEE-GHHHELDPHTWVSPHRAIQEVTNIKEQLVKL  171
S.pneumoniae        GDMLLLPGGEEEEGDH-------DHGEE-GHHHEFDPHVWLSPVRAIKLVEHIRDSLSAD  162
S.suis              DGMLLLPGSEE-GEEH-------DHSEE-GHSHAYDPHVWLSPERAITLVENIRDSLVAK  162
                        ** . .*   :.        : ..  *    ***.*:.*      . .*::.*

S.aureus            DPKHKDDYEKNYKKLNDDLKKIDNDMKQVTKDKQGNAVFISHESIGYLADCYGFVQKGIQ  236
S.haemolyticus      DPKHKNEYEKNYKKLEKSLDDIDNKLKDITKDKQGNAVFISHESLGYLADRYGFVQKGIQ  236
E.faecalis          YPKKAKTFETNAEKYLTKLTALDKEFQTALKDAKQKSFVTQHAAFGYLALDYGLKQVPIA  231
S.pneumoniae        YPDKKETFEKNAAAYIEKLQSLDKAYAEGLSQAKQKSFVTQHAAFNYLALDYGLKQVAIS  222
S.suis              YPEKKDAFETNAAAYIEKLDALDAKYSETLSAAKQKYFVTQHTAFAYLALDYGLKQVSIT  222
                     *.: . :*.*      .*  :*       .  : : .. .* :: ***  **: *  *

S.aureus            NMNA-EDPSQKELTKIVKEIRDSNAKYILYEDNVANKVTETIRKETDAKPLKFYNMESLN  295
S.haemolyticus      NMNA-EDPSQKALTQLVKEINDKNVKYILYEDNVANKVTETIRKETNAKPLKFYNMESLN  295
E.faecalis          GLTPEQEPTAGRLAELKKYVTDNQIRYIYFEKNANDKIAKTLADEANVQLEVLNPLESLT  291
S.pneumoniae        GLSPDAEPSAARLAELTEYVKKNKIAYIYFEENASQALANTLSKEAGVKTDVLNPLESLT  282
S.suis              GVAADEDPTPSRLAELTEYINKYGIKYIYFEENASKSVAETLAKETGVQLDVLNPLESLT  282
                    .:    :*:   *::: : : .    ** :*.*. . :::*: .*:..:   :  :***.

S.aureus            KEQQKKDNITYQSLMKSNIENIGKALDSGVKVKDDKAESKHDKAISDGYFKDEQVKDREL  355
S.haemolyticus      KEQSKDTSINYQTLMNKNIEALDKALDSNIKVQDDKAEHKHDKAISDGYFKDEQVKDRAL  355
E.faecalis          QKQM-DNGEDYLSVMKENLTALKKTTDTAGKEVQPETSEKTEKTVANGYFKDSEVAERTL  350
S.pneumoniae        EEDT-KAGENYISVMEKNLKALKQTTDQEGPAIEPEKA-EDTKTVQNGYFEDAAVKDRTL  340
S.suis              DEDM-KNGKDYISVMEDNLTALEKTTSQEGSEILPEEGAETAQTVYNGYFEDSAVKDRTL  341
                    .::  . .  * ::*:.*:  : :: .        :   :  ::: :***:*  * :* *

S.aureus            SDYAGEWQSVYPYLKDGTLDEVMEHKAENDPKKSAKDLKAYYDKGYKTDITNIDIKGNEI  415
S.haemolyticus      SDYEGEWQSVYPYLKNGDLDDVMKHKSEEDSSMTAKEYKAYYEKGYKTDISNIHIEGDNI  415
E.faecalis          TDYAGNWQSVYPLLKDGTLDQVFDYKAKLKKDKTPAEYKTYYDAGYQTDVDHINITDSTI  410
S.pneumoniae        SDYAGNWQSVYPFLEDGTFDQVFDYKAKLTGKMTQAEYKAYYTKGYQTDVTKINITDNTM  400
S.suis              SDYVGEWQSVYPYLLDGTLDQVWDYKAKIKGGMTAEEYKAYYDTGYKTDVDQINITDNTM  401
                    :** *:****** * :* :*:* .:*::     :  : *:**  **:**: :*.* .. :

S.aureus            TFTKDGKKHTGKYEYNGKKTLKYPKGNRGVRFMFKLVDGNDKDLPKFIQFSDHNIAPKKA  475
S.haemolyticus      TFEKNGNKVTGTYEYVGKKILDYKKGNRGVRFIYKLKNNDTPSLPKYVQFSDHNIAPKKA  475
E.faecalis          EFLVDGKPQKFTYKAAGYKILNYAKGNRGVRFLFETDDANAG-RFKYVQFSDHNIAPTKA  469
S.pneumoniae        EFVQGGQSKKYTYKYVGKKILTYKKGNRGVRFLFEATDADAG-QFKYVQFSDHNIAPVKA  459
S.suis              EFVVGDKKEKFTYKYVGYKILTYKKGNRGVRFLFEATDANAG-NYKYVQFSDHNIAPVKT  460
                     *  ..:  . .*:  * * * * ********:::  : :     *::********* *:

S.aureus            EHFHIFMGN-DNDALLKEMDNWPTYYPSKLNKDQIKEEMLAH          516
S.haemolyticus      EHFHIFMGD-NNETLLKEMDHWPTYYPASLDKDDIKEEMLAH          516
E.faecalis          AHFHIFFGGDSQESLFNEMDNWPTYYPNDLSKQEIAQEMIAH          511
S.pneumoniae        EHFHIFFGGTSQETLFEEMDNWPTYYPDNLSGQEIAQEMLAH          501
```

```
S.suis          DHFHIYFGGESQEKLLEELENWPTYYPVGLNGLEIGQEMLAH        502
                 ****::*. .:: *::*:::******  *.  :* :**:**
```

Staphylococcus aureus AdcA two zinc-binding domain and *Salmonella enterica* ZnuA single zinc-binding domain proteins sequence alignment analysis. Conserved AcdA residues of domain N-terminal (His[67], His[154], His[218], Glu[292]) involved in metal binding present in ZnuA are shown in boxes; an aspartate in ZnuA replaces glutamate [292] in AdcA, groups of strongly similar properties.

```
S.aureus        MKKKLGMLLLVPAVTLSLAACGNDDGKDKDGKVTIKTTVYPLQSFAEQIGGKHVKVSSIY     60
S.enterica      MLQKNTLL--FAA--LSAALWGSAT---QAADAAVVASLKPLGFIASAIADGVTDTQVLL     53
                * :*  :*  . *  ** *  *.     : ...:: ::: **  :*. *..  .... :

S.aureus        PAGTDIHSYEPTQKDILSASKSDLFMYTGDNLDPVAKKVASTIKDKDKKLSLEDKL-DKA     119
S.enterica      PDGASEHDYSLRPSDVKRLQGADLVVWVGPEMEAFMEKSVRNIPDNKQV-TIAQLADVKP     112
                * *:. *.*.   .*:   . :**.::.* ::: . :* . .* *:.:  :: :    *

S.aureus        KLLTDQHEHGEEHEHEGHDHEKEEHHHHHGGYDPHVWLDPKINQTFAKEIKDELVKKDPK     179
S.enterica      LLMKGADDDEDEHAHTGADEEKGDVHHHHGEYNMHLWLSPEIARATAVAIHEKLVELMPQ     172
                 *:.. .:. :** * * *.** : ***** *: *:**.*:* :: *  *:::**:  *:

S.aureus        HKDDYEKNYKKLNDDLKKIDNDMKQVTKDKQGNAVFISHESIGYLADCYGFVQKGIQNMN     239
S.enterica      SRAKLDANLKDFEAQLAATDKQVGNELAPLKGKGYFVFHDAYGYYEKHYGLTPLGHFTVN     232
                 : . : * *.:: :*   *::: :     :*:. *: *:: **  . **:.  *  .:*

S.aureus        AED-PSQKELTKIVKEIRDSNAKYILYEDNVANKVTETIRKETDAKPLKFYNMESLNKEQ     298
S.enterica      PEIQPGAQRLHEIRTQLVEQKATCVFAEPQFRPAVVEAVARGTSV---RMGTLDPLGTN-     288
                 *  *. :.* :* .:: :.:*. :: * :.   *.*:: : *..   :: .:: *..:

S.aureus        QKKDNITYQSLMKSNIENIGKALDSGVKVKDDKAESKHDKAISDGYFKDEQVKDRELSDY     358
S.enterica      IKLGKTSYSAFLSQLANQYASCLKGD----------------------------------     314
                 * .: :*.:::..  :: ...*...

S.aureus        AGEWQSVYPYLKDGTLDEVMEHKAENDPKKSAKDLKAYYDKGYKTDITNIDIKGNEITFT     418
S.enterica      ------------------------------------------------------------     314

S.aureus        KDGKKHTGKYEYNGKKTLKYPKGNRGVRFMFKLVDGNDKDLPKFIQFSDHNIAPKKAEHF     478
S.enterica      ------------------------------------------------------------     314

S.aureus        HIFMGNDNDALLKEMDNWPTYYPSKLNKDQIKEEMLAH       516
S.enterica      --------------------------------------       314
```

Staphylococcus aureus AdcA two zinc-binding domain and *Salmonella enterica, Escherichia coli, Bacillus subtilis* ZinT proteins sequence alignment analysis. Conserved AdcA residues of C-terminal domain (His^{468}, His^{477}, His^{479}) involved in metal binding and present in ZinT are shown in boxes.

```
S.aureus        MKKKLGMLLLVPAVTLSLAACGNDDGKDKDGKVTIKTTVYPLQSFAEQIGGKHVKVSSIY      60
B.subtilis      ------------------------------------------------------------      0
E.coli          ------------------------------------------------------------      0
S.enterica      ------------------------------------------------------------      0

S.aureus        PAGTDLHSYEPTQKDILSASKSDLFMYTGDNLDPVAKKVASTIKDKDKKLSLEDKLDKAK      120
B.subtilis      ------------------------------------------------------------      0
E.coli          ------------------------------------------------------------      0
S.enterica      ------------------------------------------------------------      0

S.aureus        LLTDQHEHGEEHEHEGHDHEKEEHHHHHGGYDPHVWLDPKINQTFAKEIKDELVKKDPKH      180
B.subtilis      ------------------------------------------------------------      0
E.coli          ------------------------------------------------------------      0
S.enterica      ------------------------------------------------------------      0

S.aureus        KDDYEKNYKKLNDDLKKIDNDMKQVTKDKQGNAVFISHESIGYLADCYGFVQKGIQNMNA      240
B.subtilis      ------------------------------------------------------------      0
E.coli          ------------------------------------------------------------      0
S.enterica      ------------------------------------------------------------      0

S.aureus        EDPSQKELTKIVKEIRDSNAKYILYEDNVANKVTETIRKETDAKPLKFYNMESLNKEQQK      300
B.subtilis      ----------------------------------MKISFATRLGVLTFGSLLVLAGCQAS      26
E.coli          ----------------------------------MAI---------RLYKLAVALGVFI-      16
S.enterica      ----------------------------------MVI---------HLKKLTMLLGMLL-      16
                                                    *          : .:

S.aureus        -----KDNITYQSLMKSNIENIGKALDSG-VKVKDDKAESKHDKAISDGYFKDEQVKDRE      354
B.subtilis      GSSKGESHKTSSSVAEDASKTQEQSLGSHDHEHDHSHAHDEETEKIYEGYFKNSQVKDRP      86
E.coli          --------------------VSAPAFSHG--HHSHGKPLTEVEQKAANGVFDDANVQNRT      54
S.enterica      --------------------VNSPAFAHG--HHAHGAPMTEVEQKAAAGVFDDANVRDRA      54
                                        ::     .  ..    :  :    * *.: :*::*

S.aureus        LSDYAGEWQSVYPYLKDGTLDEVMEHKAENDPKKSAKDLKAYYDKGYKTDITNIDIKGNE      414
B.subtilis      LTDWEGDWQSVYPYLQDGTLDEVFSYKSKHEGDKTAEEYKEYYKKGYKTDVDRIFIQKDT      146
E.coli          LSDWDGVWQSVYPLLQSGKLDPVFQKKADADKTKTFAEIKDYYHKGYATDIEMIGIEDGI      114
S.enterica      LTDWDGMWQSVYPYLVSGELDPVFRQKAKKDPEKTFEDIKAYYRKGYVTNVETIGIENGV      114
                *:*: * ****** * .* ** *:  *:. :  *:  : * ** *** *::  * *: .

S.aureus        ITFTKDGKKHTGKYEYNGKKTLKYPKGNRGVRFMFKLVDGNDKDLPKFIQFSDHNIAPKK      474
B.subtilis      VTFFKNGKEYSGKYTYDGYEILTYDAGNRGVRYIFKRAK-EAEGLPQYIQFSDHSIDPTK      205
E.coli          VEFHRNNETTSCKYDYDGYKILTYKSGKKGVRYLFECKDP-ESKAPKYIQFSDHIIAPRK      173
S.enterica      IEFHRDNNVASCKYNYAGYKILTYASGKKGVRYLFECKDA-NSKAPKYVQFSDHIIAPRK      173
                : * ::.:  : ** * * : *.*  *::***::*:  .   .  *:::***** * * *

S.aureus        AEHFHIFMGNDN-DALLKEMDNWPTYYPSKLNKDQIKEEMLAH  516
B.subtilis      AGHYHLYWGDDR-KALLDEVKNWPTYYPSEMDGHDIAHEMMAH  247
E.coli          SSHFHIFMGNDSQQSLLNEMENWPTYYPYQLSSEEVVEEMMSH  216
S.enterica      SAHFHIFMGNTSQQALLQEMENWPTYYPYQLKANEVVDEMLHH  216
                : *: :: *:   .:**.*:.******* ::. .:: .**: *
```
